# Religious Consolation to the Insane

**Published:** 1855-10-01

**Authors:** Jos. Souter

**Affiliations:** Chaplain to the Essex County Lunatic Asylum.


### RELIGIOUS CONSOLATION TO THE INSANE.

BY THE REV. JOS. SOUTER,

*Chaplain to the Essex County Lunatic Asylum.*

As might be expected in an asylum for the insane, a considerable number of the inmates are not capable, in any degree perceptible to us, of being influenced by religious or any other teaching. I speak of those who from birth have been insane, and of those whom disease has reduced to a state of dementia. But there are others upon whose minds the ministrations of religion produce a strong and marked impression. I cannot forbear here to refer to one or two examples. One is that of a male patient who was restored to sanity. Dr. Campbell informed him that he would recommend his discharge. He was most grateful for the kind intention, but begged to be permitted to remain, as he felt that he had not many weeks to live. He said he knew that if he left the asylum he could not enjoy the advantage of medical advice, and of other kindnesses that he received here; but more than all, he should regret to be taken away from the daily services of the chapel, which had been so great a comfort and blessing to him. Dr. Campbell was moved by the man's entreaties, and with great kindness permitted him to remain, though he knew that by so doing it would only add to the number of deaths. The religion which that man so highly prized supported him through the brief remainder of his days. He looked forward to his end with calmness, and met it with the faith and resignation of a Christian. A rapid consumption, in a few weeks, carried him beyond the reach of all earthly sorrows and trouble, we trust, "to another and a better world."

A second example is that of a female patient, who has now left the asylum in a state of convalescence. She was upwards of sixty years of age, but had never partaken of the Holy Communion till she received it in the asylum chapel. From her manner of speaking on this subject, I should judge that she was strongly impressed with religious feeling. She, too, had found the services of the church to be a blessing to her. I trust she has carried with her to her home that principle of piety which shall be a strength and consolation to her for the remaining years of her life, and which shall enable her, when death comes, to triumph over it, through faith in Him who "has opened to all believers" the gate of everlasting life.

There are not a few patients who, from mistaken notions of religion, refuse the consolations which it offers them, and cling with a strange tenacity to the belief that, though there may be forgiveness for all others, there is none for them. You may quiet their terrors of conscience one day, and on the next you shall find they have returned again to their old and cherished despair. But even of cases such as these I have seen some, not able indeed totally to subdue their melancholy, but strengthened to bear it through confidence in God. I have seen many whose despondency has vanished entirely, and all the gloomy fancies that had haunted them night and day for years have been dispelled; but that change has been the precursor of death. One especially I remember, who died at the commencement of the year. Her dread of death, when first she felt its advances, her ravings of despair, her firm persuasion that she had committed the "unpardonable sin," were sometimes fearful to witness. But a few days before she died all was changed, and happily changed, not in a return to those high delusions which she had once been taught to call religion, but changed to a calm, humble, and penitent belief in the atonement. There was no ecstasy, no unbounded rapture, but there was repentance, resignation, faith. It is not at all unusual in certain similar cases to see them, a few days before death, lighted up with new hopes. The mind is freed from its terrors and its delusions. The sunshine of earlier days revisits the soul. Allow me, however, here to guard against an impression that this is always the case. Many of the insane die as they have lived. Their minds are not lighted up with even a momentary flash of returning intelligence. I have had the most abundant opportunity afforded me for forming this judgment, for I have visited the sick daily. I have gone to the bedside of the dying, in every case, thinking it possible that even in the most bewildered and lost there might occur some moment before death, in which the mind would be prepared to receive the comfort and instruction of religious truth. Even during the prevalence of cholera, feeling it to be my duty to keep up this practice, I went every morning to the bedside of the patients upon whom the scourge of that mysterious disease had fallen. And I rejoice to say, that terrible as it was to witness so many smitten as it were by an invisible hand,—cold and dead in a few hours,—my visits were sometimes attended also with circumstances of a most affecting and not unpleasing nature. The majority, indeed, sunk at once into total unconsciousness or apathy. But there were some actively alive to their danger, and anxious to receive the last consolations of religion. I cannot omit to notice one in particular. He was an old sailor; he lived several days after his first attack. Each day as I entered the ward he lifted up his hands, and placed them together in an attitude of devotion, as if to signify to me a request to pray with him. I asked him if that were his wish, and he murmured, as well as he was able, "Yes." I then read a few verses from the Bible, and knelt down at his bedside to pray for pardon and strength, and to commend his soul to God; and never have I heard a more earnest and devout amen than that which was whispered so faintly, but so fervently, by that dying man. I have not often witnessed a scene more touching. In that one room were the beds of four other dying men. The eyes of more than one there were filled with tears. Let us trust that He, "who despiseth not the sighing of a contrite heart, nor the desire of such as be sorrowful," would mercifully regard those tears, and hear those prayers, and receive those afflicted men into everlasting rest.—*From the recent Official Report of the Asylum.*

---

